# Mitofusin 2 Protects Hepatocyte Mitochondrial Function from Damage Induced by GCDCA

**DOI:** 10.1371/journal.pone.0065455

**Published:** 2013-06-06

**Authors:** Yongbiao Chen, Lizhi Lv, Zhelong Jiang, Hejun Yang, Song Li, Yi Jiang

**Affiliations:** Department of Hepatobiliary Surgery, Fuzhou General Hospital, Fuzhou, China; Nihon University School of Medicine, Japan

## Abstract

Mitochondrial impairment is hypothesized to contribute to the pathogenesis of chronic cholestatic liver diseases. Mitofusin 2 (Mfn2) regulates mitochondrial morphology and signaling and is involved in the development of numerous mitochondrial-related diseases; however, a functional role for Mfn2 in chronic liver cholestasis which is characterized by increased levels of toxic bile acids remain unknown. Therefore, the aims of this study were to evaluate the expression levels of Mfn2 in liver samples from patients with extrahepatic cholestasis and to investigate the role Mfn2 during bile acid induced injury in vitro. Endogenous Mfn2 expression decreased in patients with extrahepatic cholestasis. Glycochenodeoxycholic acid (GCDCA) is the main toxic component of bile acid in patients with extrahepatic cholestasis. In human normal hepatocyte cells (L02), Mfn2 plays an important role in GCDCA-induced mitochondrial damage and changes in mitochondrial morphology. In line with the mitochondrial dysfunction, the expression of Mfn2 decreased significantly under GCDCA treatment conditions. Moreover, the overexpression of Mfn2 effectively attenuated mitochondrial fragmentation and reversed the mitochondrial damage observed in GCDCA-treated L02 cells. Notably, a truncated Mfn2 mutant that lacked the normal C-terminal domain lost the capacity to induce mitochondrial fusion. Increasing the expression of truncated Mfn2 also had a protective effect against the hepatotoxicity of GCDCA. Taken together, these findings indicate that the loss of Mfn2 may play a crucial role the pathogenesis of the liver damage that is observed in patients with extrahepatic cholestasis. The findings also indicate that Mfn2 may directly regulate mitochondrial metabolism independently of its primary fusion function. Therapeutic approaches that target Mfn2 may have protective effects against hepatotoxic of bile acids during cholestasis.

## Introduction

Cholestasis is characteristic of the most common and serious liver diseases, could be caused by conditions that the enterohepatic circulation is interrupted and bile acids accumulate within the liver [1]. The pathological features of cholestasis, namely inflammatory cell infiltration, hepatocyte necrosis, and liver fibrosis, are eventually followed by cirrhosis [2], [3]. Early intervention is a key factor in preventing the progression of cholestatic liver disorders. There is increasing evidence that mitochondria play crucial roles in the pathogenesis of chronic liver cholestasis. For example, our previous studies showed that hepatic mitochondrial energy and the mtDNA copy number level progressively decrease in patients with extrahepatic cholestasis [4]. GCDCA is the main toxic component of bile acid in patients with extrahepatic cholestasis [3], [5], [6]. Multiple lines of evidence have indicated that GCDCA disrupt the electron transfer chain, increase the reactive oxygen species (ROS) levels, and contribute to mitochondrial damage [7], [8], [9], [10].

Recently, mitochondria have been proven to be highly dynamic organelles that undergo constant fission and fusion, and the balance of these opposing processes regulates the morphology and normal function of mitochondria [11], [12], [13], [14], [15]. Emerging evidence indicates that mitochondrial metabolism is regulated through the manipulation of the proteins involved in mitochondrial dynamics, particularly the Mfn2 protein. Mfn2 is a transmembrane GTPase that is embedded in the outer mitochondrial membrane and is widely expressed in the liver, the heart, and other organs [11]. Changes in Mfn2 activity are linked to various human mitochondria-associated diseases, such as Charcot-Marie-Tooth type 2A neuropathy, diabetes, and cardiovascular diseases [16], [17], [18], [19], [20]. Mfn2 insufficiency and the subsequent disruption of mitochondrial dynamics contribute to the development of mitochondrial membrane permeabilization, the loss of the inner mitochondrial membrane potential, and cell apoptosis. In addition, Mfn2 participates in various cell signaling cascades, some of which are thought to extend beyond the function of mitochondrial fusion. The effects of Mfn2 may be attributed to the direct regulation of cell respiration, substrate oxidation, and glucose oxidation [11], [21].

In light of the profound impact of Mfn2 on mitochondria function, exploring the mechanism underlying the function of Mfn2 in extrahepatic cholestasis is an important area of clinical research. In this study, we first investigated the expression levels of Mfn2 in samples from patients with extrahepatic cholestasis and in the hepatocyte cell line L02 treated with GCDCA. We then investigated the effects of Mfn2 on mitochondrial metabolism in liver tissue from patients with extrahepatic cholestasis and the possible protective effects of Mfn2 overexpression in the L02 cell lines.

## Materials and Methods

### Patients and Methods

The subjects in this study consisted of 14 patients who were admitted to the Surgery Department due to an obstructive jaundice. In these patients, obstructive jaundice was the result of pancreatic cancer in 8 patients, a periampullary tumor in 4 patients, and cholangiocarcinoma in 2 patients. Liver tissue samples were obtained during major non-hepatic abdominal surgery. The laboratory studies included serum liver tests (alanine aminotransferase, aspartate aminotransferase, γ-glutamyl transpeptidase, alkaline phosphatase, total bilirubin, and total bile acid levels), hepatitis B and C serology (hepatitis B surface antigen, antibody to hepatitis B surface antigen, antibody to hepatitis B core antigen, and serum hepatitis C virus RNA), autoimmune serology (antimitochondrial antibody and antinuclear antibody), HDL– cholesterols, LDL- cholesterols and TG. All of the serum specimens were collected during the morning of the operation day. Control liver tissue (control group, n = 12) was obtained from non-jaundiced patients with a pancreatic tumor (n = 5) and from patients undergoing cholecystectomy for gallstones (n = 7). All of the subjects included in the study were negative for viral hepatitis infection, liver autoimmune disorders, and metabolic disorders and were not being treated with hepatotoxic drugs. The patients who presented with recent alcohol abuse were also excluded. The characteristics of these patients are summarized in Table 1. All research involving human participants in this manuscript have given written informed consent (as outlined in PLOS consent form), and the study was approved by the ethics statement of Fuzhou General hospital and was carried out in accordance with the provisions of the Declaration of Helsinki. After collection, the liver samples were immediately immersed in liquid nitrogen and stored at −80°C until processing for the biochemical assays.

**Table 1:** Clinical characteristics of the patients.

	Control	Cholestatic	p value
Male/female	5/7	8/6	>0.05
Age (years)	51.5±10.2	48.5±7.0	>0.05
Alanine aminotransferase (U/L)	30.5±18.5	198.4±110.8	<0.001
Aspartate aminotransferase (U/L)	30.8±19.5	149±56.7	<0.001
Alkaline phosphatase (U/L)	87.1±40.6	516.5±178	<0.001
γ_Glutamyl transpeptidase (U/L)	50.5±46.7	618.2±351	<0.001
Total bilirubin (umol/L)	14±3.1	168.5±62.0	<0.001
Direct bilirubin (umol/L)	5.1±3.8	90.8±39.2	<0.001
Total bile acid (umol/L)	3.7±1.8	145±76.1	<0.001
HDL - cholesterols(mmol/L)	0.83±0.37	0.75±0.32	>0.05
LDL- cholesterols(mmol/L)	2.35±0.62	2.53±0.54	>0.05
TG(mmol/L)	1.31±0.45	1.27±0.38	>0.05

### Cell Culture and Treatment

The human normal hepatocyte cell line L02 was purchased from the cell bank of the Institute of Biochemistry and Cell Biology (Shanghai, China). The L02 cells were cultured in RPMI 1640 medium (Invitrogen Corp., Carlsbad, CA, USA) supplemented with 10% heat-inactivated fetal calf serum (HyClone, Logan, UT, USA) and 1% v/v penicillin/streptomycin (Sigma-Aldrich, St. Louis, MO, USA) in a 5% CO_2_ humidified atmosphere at 37°C. The L02 cells were grown to 80% confluence and then exposed to various final concentrations of GCDCA (25, 50, 75, or 100 µM for 6 h [4], [6], [22]. The GCDCA (Sigma-Aldrich) was dissolved in sterile phosphate-buffered saline (PBS) to produce a 25-mM stock solution and then used to produce serial dilutions in the cell culture medium before application.

### Histological Analysis

The formalin fixed liver samples were embedded in paraffin and sectioned, and then stained with hematoxylin and eosin for observation under a light microscope (Olympus IX71, Japan), as the method previously described [23].

### Oxidative Stress Determination

The oxidative stress in the liver tissue samples was measured using a Lipid Peroxidation MDA Assay Kit (Beyotime Co. Shanghai, China) according to the manufacturer’s instructions. Intracellular ROS levels were determined using a DCFH-DA Assay Kit (Beyotime, Shanghai, China). Briefly, after the indicated treatments, cells cultured in 96-well plates were incubated in the dark using DCFH-DA for 30 min at 37°C. After gently mixing the solutions, the intensity readings for the above mixtures were measured using an Infinite™ M200 Microplate Reader (Tecan, Mannedorf, Switzerland).

### ATP Content Determination

The ATP level of the liver tissue cells was measured using an ATP Determination Kit (Beyotime Co. Shanghai, China). The ATP concentrations were analyzed using an Innite M200 microplate reader (Tecan, Mannedorf, Switzerland) according to the method previously described [24].

### Measurement of ΔΨm

The ΔΨm was determined using the Mitochondrial Membrane Potential Assay Kit and JC-1 (Invitrogen Corp., Carlsbad, CA, USA). After the indicated treatments, the cells were loaded with 1×JC-1 in growth medium at 37°C for 20 min. The resultant green and red fluorescence intensities were detected using an Infinite™ M200 Microplate Reader (Tecan, Mannedorf, Switzerland). The ΔΨm measurements of cells in each treatment group were calculated as the ratio of red to green fluorescence.

### Cell Viability Assay

Cell viability was determined using the Cell Counting Kit-8 (CCK-8) (Dojindo Laboratories, Japan) according to the manufacturer’s instructions. Briefly, cells were seeded onto 96-well microtiter plates at a density of 5×10^3^ cells/well. After being treated with GCDCA, the cells were incubated with 10 µl of CCK-8 solution for 2 h. The absorbance was detected at 450 nm using a model ElX800 microplate reader (Bio-Tek Instruments).

### Assessment of Mitochondrial Morphology

L02 cells were stained using 100 nM MitoTracker Red CMXRos probe for 30 min at 37°C according to the manufacturer's instructions, and the mitochondrial morphology was observed under a Leica confocal laser scanning microscope (TCS SP2, Germany). The fragmented mitochondria and filamentous mitochondria were identified as previously described [25], [26]. For quantitative analysis of the changes in mitochondrial morphology, the proportions of cells with fragmented mitochondrial patterns were recorded in at least 50 cells per cover slip observed on adjacent fields at a magnification of 63x. We performed the experiments using a “blind” counter.

### Western Blotting

The liver tissues and L02 cells were lysed using RIPA buffer (Beyotime Co. Shanghai, China) in the presence of a cocktail of protease inhibitors (Roche, Indianapolis, IN, USA) and then centrifuged at 12,000 g for 30 min at 4°C. Electrophoresis on a 10% standard SDS-polyacrylamide gel was used to separate 30 µg of total protein; the proteins were then transferred to nitrocellulose membranes (Bio-Rad). After the nitrocellulose membranes were blocked, they were incubated with mouse anti-Mfn2 antibody (Cat. No ab56889, Abcam, Cambridge, MA, USA) and mouse anti-human β-actin antibody (Santa Cruz Biotechnology, Santa Cruz, CA, USA) overnight at 4°C. After 3–4 washes, the membranes were incubated with IRDye800CW-labeled (Li-Cor, Lincoln, NE, USA) donkey anti-mouse antibody. The fluorescent signals were detected and quantified using an Odyssey infrared imaging system.

### Real-time PCR Analysis to Detect the Mfn2 mRNA Expression

MtDNA copy number was detected in the iQ5 Real-Time PCR Detection System (Bio-Rad, Hercules, CA,USA) with the SYBR Green I detection method, as previously described. [4] Mfn2 (5′-ATGCATCCCCA CTTAAGCAC-3′) and (5′-CCAGAGGGCAGAACTTTGTC-3′); G-APDH (5′-TGACAACAGCCTCAAGAT-3′) and (5′-GAGTCCTTCCACG ATA CC-3′).

### Plasmid Construction and Transfection

A plasmid targeted to human Mfn2 and a truncated Mfn2 mutant (hMfn2D602–757) were designed by the Invitrogen Corporation (Shanghai, China) and cloned into the pcDNA™6.2-GW/EmGFPmiR vector. The negative controls were also provided by the Invitrogen Corporation. The plasmids were transfected into L02 cells using Optimum-Minimum Essential Medium with Lipofectamine 2000 (Invitrogen, Rockville, MD, USA) according to the manufacturer’s instructions.

### Statistics

All of the experimental data are expressed as the means ± SEM, and each experiment was performed at least three times. Significance was evaluated by Fisher’s exact probability test or T test in Table 1. The data comparisons between the groups were performed using a one-way ANOVA, and P<0.05 was considered to be statistically significant.

## Results

### Mitochondrial Dysfunction and Loss of the Mitochondrial Fusion Protein Mfn2 after Liver Injury in Patients with Extrahepatic Cholestasis

**Figure 1:**
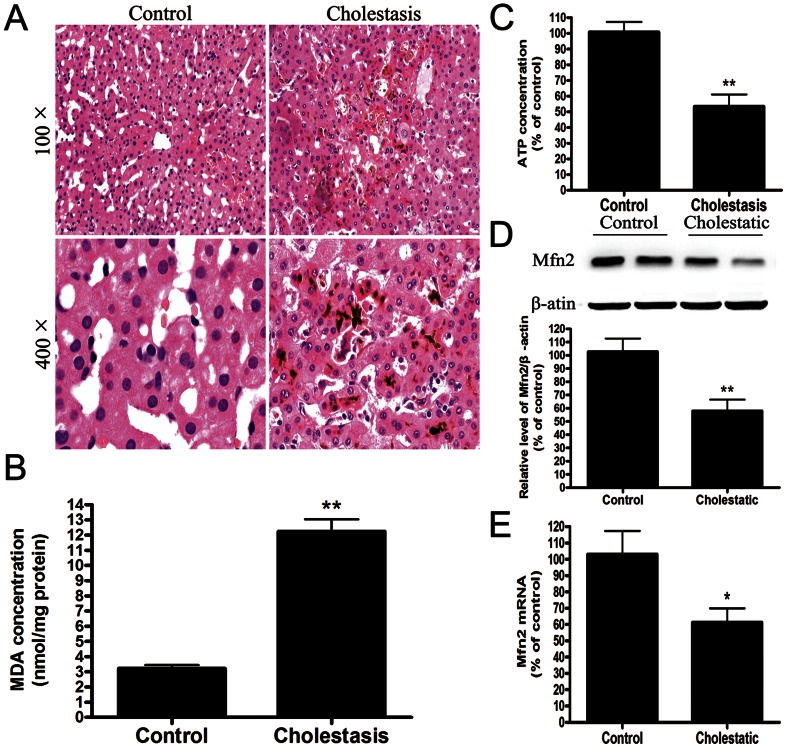
Mitochondrial dysfunction and loss of the mitochondrial fusion protein Mfn2 in patients with extrahepatic cholestasis. (A) Representative histology of human liver tissue. (B) The MDA concentrations and (C) ATP concentrations in human liver tissue were determined using a MDA Determination Kit and an ATP Determination Kit. (D) Representative immunoblot and quantification analysis of the protein level of Mfn2 (86 kDa) in human liver tissue. β-actin (42 kDa) was the internal standard for protein loading. The results are expressed as a percentage of the control value, which was set at 100%. The values are the means ± SEM. *P<0.05 versus the control group.

The clinical features of the patients are summarized in Table 1. There were no significant case-control differences in the distributions of age, group, or gender. The key clinical factors, including the total bile acid, alanine aminotransferase, aspartate aminotransferase, alkaline phosphatase, γ-glutamyl transpeptidase, and bilirubin levels, were significantly higher in the patients compared with the controls. Light microscopy revealed the presence of hepatic cord disturbance and intracellular cholestasis in the cholestatic patients, and focal necrosis and inflammatory cell infiltration were observed (Fig. 1A). In addition, the MDA levels were significantly increased by 400% in the livers of the patients (Fig. 1B). In contrast, ATP production was significantly decreased (Fig. 1C). In parallel with this finding, the western blot analysis revealed that the protein levels of Mfn2 were significantly decreased by 60% in the patients compared with the negative controls as well as the decrease of the Mfn2 mRNA. (Fig. 1D and 1E).

### Reduction of Mfn2 Expression and the Impairment of Mitochondrial Function in GCDCA Treated L02 Cells

**Figure 2:**
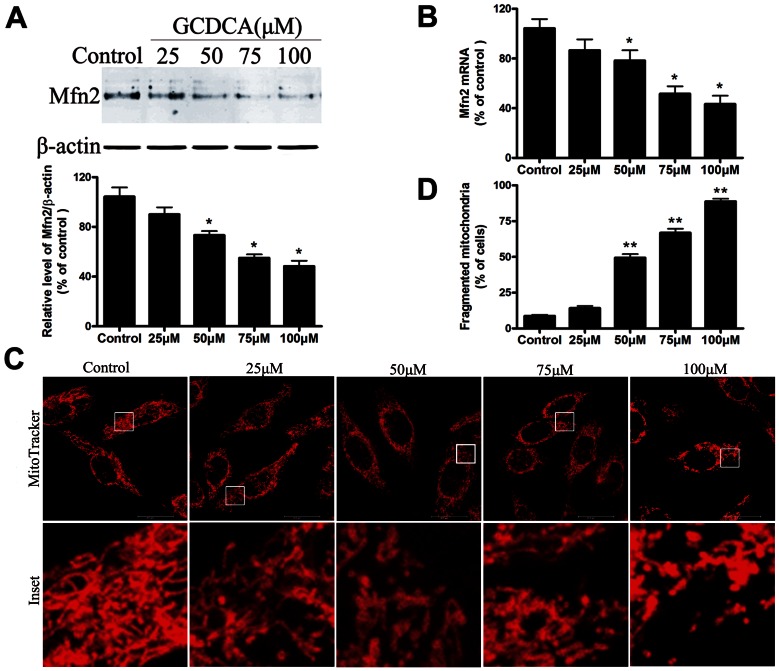
GCDCA decreased mitochondrial fusion protein Mfn2 expression and induced mitochondria fragmentation in the treated L02 cells. (A) Representative immunoblot and quantification analysis of the protein level of Mfn2 (86 kDa) in the L02 cells. β-actin (42 kDa) was the internal standard for protein loading. (B) The specific fluorescence probe, MitoTracker Red CMXRos (red), was used to detect changes in the mitochondrial morphology. After the L02 cells were treated with various doses of GCDCA, representative changes in the mitochondrial morphology were detected under confocal microscopy. (C) Proportions of cells with fragmented mitochondrial pattern was determined in at least six different cultures under basal conditions (untreated), and after the L02 cells were treated with various doses (25, 50, 75, or 100 µM) of GCDCA for 6 h. The results are expressed as a percentage of the control value, which was set at 100%. The values are the means ± SEM. *P<0.05 versus the control group, n = 6.

**Figure 3:**
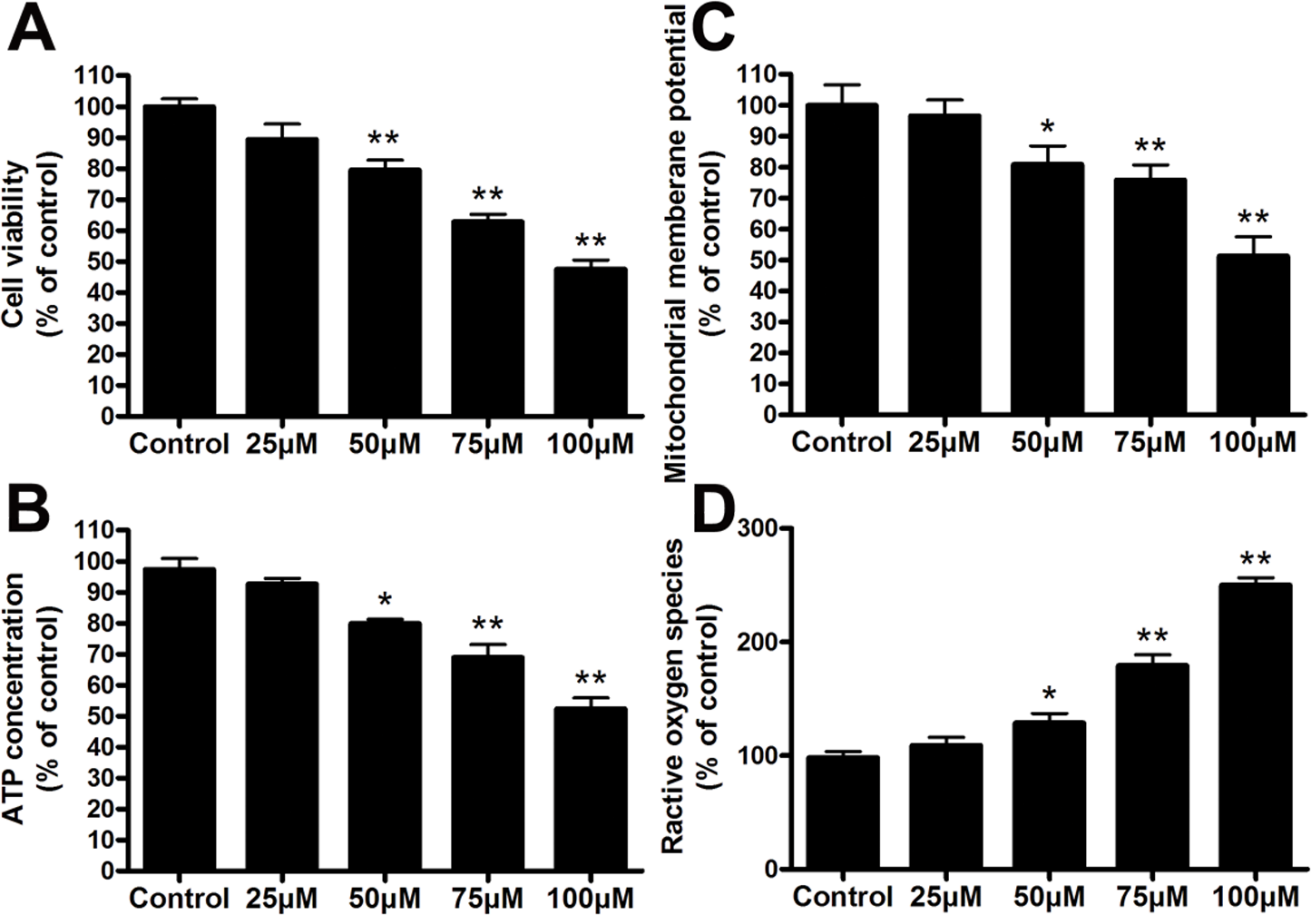
GCDCA-induced impairment of mitochondrial function in the treated L02 cells (A) Cell viability, (B) ATP concentrations, (C) ΔΨm, and (D) ROS production were assayed. The L02 cells were treated with various doses (25, 50, 75, or 100 µM) of GCDCA for 6 h. The results were expressed as a percentage of the control value, which was set at 100%. The values are the means ± SEM. *P<0.05 versus the control group, n = 6.

To explore the mechanism of bile acid-induced hepatotoxicity during extrahepatic cholestasis, we treated L02 cells with GCDCA and then assayed the Mfn2 expression and mitochondrial function. The effect of GCDCA on endogenous Mfn2 expression was then confirmed by analyzing the protein level in L02 cells using Western blotting. As a result, after cells were treated with 25, 50, 75, or 100 µM GCDCA for 6 h, the protein levels of Mfn2 were significantly decreased by 1.7-, 1.96-, 2.51-, and 2.62-fold, respectively, compared with the controls (Fig. 2A). Moreover, the levels of Mfn2 mRNA decreased in a similar pattern (Fig. 2B). Consistent with the decreases in the levels of Mfn2, GCDCA induced a significant dose-dependent increase in the number of fragmented mitochondria with ring-shaped structures, as determined by confocal microscopy of the MitoTracker-stained L02 cells (Fig. 2C). In addition, treating cells with various concentrations of GCDCA decreased cell viability approximately 9, 18, 33, and 55% (Fig. 3A). As expected, these reductions were accompanied by decreased ATP levels (Fig. 3B) and ΔΨm values (Fig. 3C), as well as the up regulation of ROS production in a time-dependent manner (Fig. 3D). Because the important role of Mfn2 in mitochondrial metabolism and the decrease of Mfn2 lever in GCDCA-treated L02 cells, we speculate that GCDCA may cause L02 cells mitochondrial dysfunction through the decreased expression of Mfn2.

### The Overexpression of Mfn2 Attenuated GCDCA-induced Mitochondrial Dysfunction and Restored the Mitochondrial Morphology

**Figure 4:**
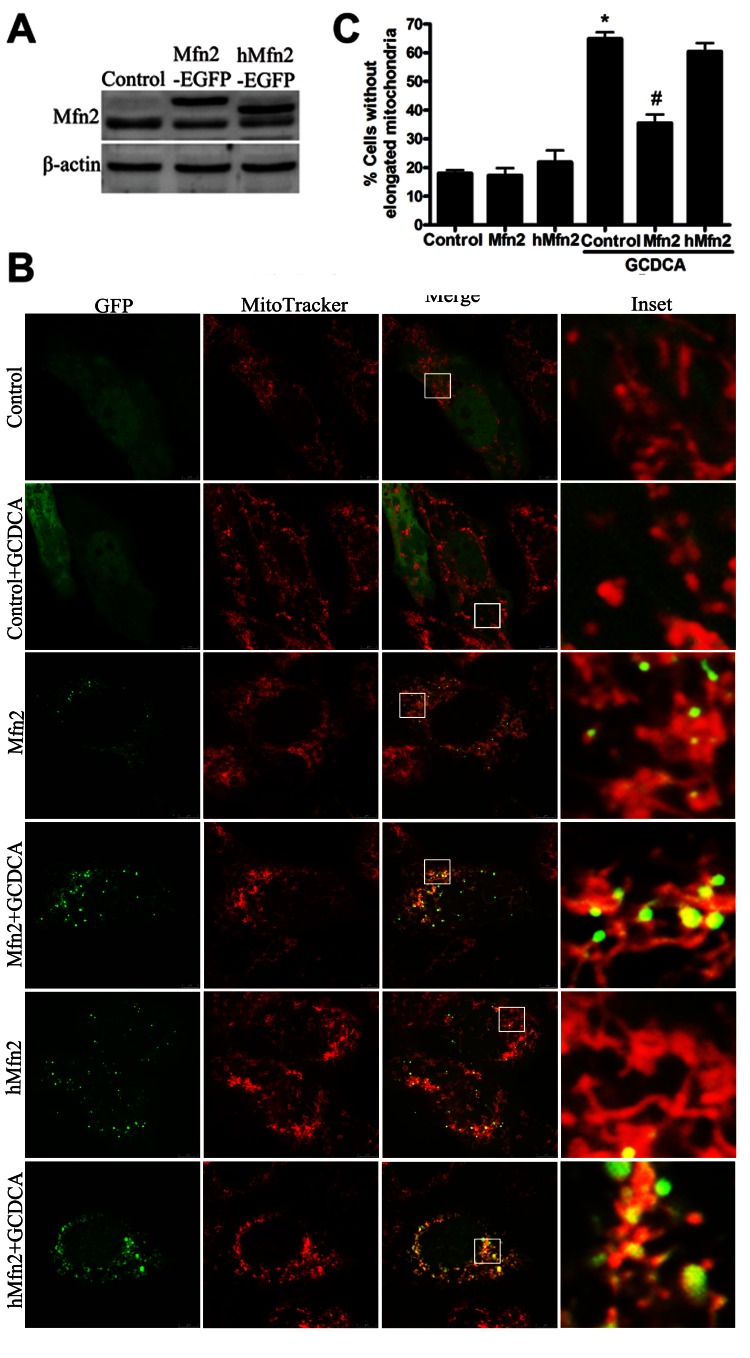
Effect of the overexpression of Mfn2 or the truncated Mfn2 mutant on mitochondrial morphology and mitochondrial function in GCDCA-treated L02 cells. (A) Western blot analysis was used to detect the expression of Mfn2-GFP and the truncated Mfn2 mutant transfected into L02 cells. (B) Confocal microscope photographs indicated increased mitochondrial localization of Mfn2 and the truncated Mfn2 mutant and their roles in mitochondrial morphology. (C) Proportions of cells with fragmented mitochondrial pattern was determined in at least six different cultures under basal conditions (untreated), and after the L02 cells were treated with various doses (25, 50, 75, or 100 µM) of GCDCA for 6 h. The results are expressed as a percentage of the control value, which was set at 100%. The values are the means ± SEM. *P<0.05 versus the control group, **^#^**P<0.05, **^##^**P<0.01 versus the GCDCA group, n = 6.

To assess whether the restoration of Mfn2 expression is sufficient to enhance mitochondrial activity in GCDCA-induced hepatotoxicity, we overexpressed Mfn2 in L02 cells by transient transfection. Mfn2 overexpression efficiently reversed the decrease in cell viability in L02 cells that had been treated with 75 µM GCDCA for 6 h (Fig. 4C). In parallel, Mfn2-overexpressing cells exhibited marked increases in ATP (Fig. 4D) and ΔΨm (Fig. 4E) levels and a decrease in the rate of ROS production (Fig. 4F). Notably, overexpressed Mfn2 efficiently reversed the increase in mitochondrial fragmentation in the transfected L02 cells that had been incubated with GCDCA (Fig. 4B).

### The Protective Effect of Mfn2 is Independent, at Least in Part, of its Role as a Mitochondrial Fusion Protein in GCDCA-treated L02 Cell Lines

**Figure 5:**
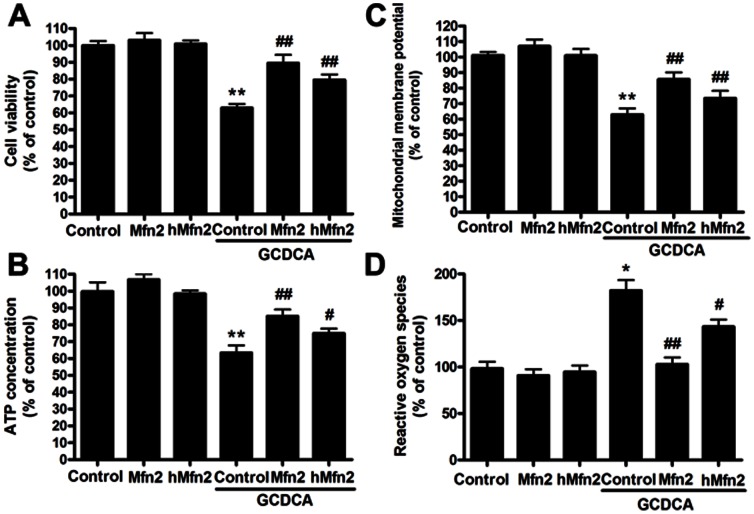
Effect of the overexpression of Mfn2 or the truncated Mfn2 mutant on mitochondrial function in GCDCA-treated L02 cells. The overexpression of Mfn2 or truncated Mfn2 (A) protected cell viability in L02 cells, (B) reversed the reduction in ATP levels, (C) ameliorated the decrease in ΔΨm, and (D) reduced the excess ROS production following treatment with GCDCA. The results are expressed as a percentage of the control value, which was set at 100%. The values are the means ± SEM. *P<0.05 versus the control group, **^#^**P<0.05, **^##^**P<0.01 versus the GCDCA group, n = 6.

Previous studies have demonstrated that Mfn2 overexpression affects the morphology of mitochondrial filaments and restores mitochondrial function. To determine whether the effects of Mfn2 on mitochondrial metabolism could be a consequence of mitochondrial fusion, we generated a truncated Mfn2 mutant (hMfn2D602–757) that lacked the amino acid residues from 602 to 757 and therefore lacked the transmembrane domain and the normal C-terminal end. In the present study, confocal microscopy revealed that most of the truncated Mfn2 was observed in the mitochondria, similar to the localization of wild-type Mfn2. However, the overexpression of truncated Mfn2 in the L02 cells did not block the mitochondrial fragmentation induced by GCDCA (Fig. 4B and 4C). Compared with wild-type Mfn2, truncated Mfn2 remained effective in reversing mitochondrial dysfunction in GCDCA-treated L02 cells (Fig. 5A, B, C, D).

## Discussion

To our knowledge, this study is the first evaluate the relationship of Mfn2 in extrahepatic cholestasis. We demonstrated that endogenous Mfn2 expression is significantly reduced in extrahepatic cholestatic patients. Furthermore, we showed that GCDCA stimulation decreases Mfn2 expression in L02 cells in a manner that is similar to the decreased expression detected in patients with extrahepatic cholestasis, indicating that the liver changes seen in the patients are a genuine reflection of the changes that occurred in the L02 cells. Notably, Mfn2 overexpression not only profoundly changed the mitochondrial network morphology but also markedly ameliorated the mitochondrial dysfunction in GCDCA-treated L02 cells. Most importantly, a C-terminal truncated form of Mfn2 with no capacity to induce mitochondrial fusion was equally able to protect against the hepatotoxicity of GCDCA. Based on the above findings, Mfn2 may act as an important factor in the pathogenesis of extrahepatic cholestasis.

Chronic liver cholestasis is responsible for the rapid development of progressive liver failure, for which there is still no effective therapy. Experimental evidence indicates that mitochondrial dysfunction is crucial in the pathogenesis of liver cholestasis [27], [28]. Recently, a growing body of evidence indicates that mitochondrial fusion and fission have important roles in establishing, maintaining, and remodeling mitochondrial function. The abnormalities in mitochondrial dynamics are associated with neurodegenerative diseases, cardiovascular disorders, and diabetes mellitus [29], [30], [31]. In the present study, GCDCA stimulated morphological changes in the mitochondria of L02 cells from the characteristic long shape to a round and short shape, which may suggest that defects in organelle fusion and fission are important in extrahepatic cholestasis.

Mfn2 is an integral outer mitochondrial membrane protein [32]. Both the NH2- and COOH-terminal parts are exposed to the cytosol and a small part of Mfn2 presumably faces the intermembrane space, and splits the transmembrane domain into two parts [33]. Although it has been shown that the expression of Mfn2 was down-regulated in liver diseases [17], [34], [35], the role of Mfn2 in chronic cholestatic liver diseases has not been investigated. In this study, we showed that GCDCA (main component of bile acid) down-regulated Mfn2 expression in patient in vivo and L02 cells in vitro. We hypothesize that the pathogenesis of chronic cholestatic liver diseases are related to mfn2 expression.

Mitochondrial morphology is regulated by fusion and fission processes that are controlled by a growing set of “mitochondria-shaping” proteins, particularly Mfn2. Mfn2 plays an important role in mitochondria fusion and is critical for mitochondrial function. Growing evidence indicates that increased Mfn2-dependent mitochondrial fusion serves to maintain a tubular mitochondrial network and to optimize mitochondrial function. For example, Mfn2 suppression leads to the fragmentation of the mitochondrial network and is associated with decreased mitochondrial membrane potential and defects in ATP synthesis, whereas the induction of Mfn2 increases glucose oxidation and restores ATP levels [36], [37], [38]. In addition, the ablation of Mfn2 led to a disruption of the mitochondrial network and an increase in ROS production [13], [39]. Our results indicate that Mfn2 overexpression increased mitochondrial fusion, followed by the reversal of mitochondrial dysfunction, such as the reduction of the excess ROS production, reversal of the reduction in ATP levels, and amelioration of the decrease in ΔΨm caused by GCDCA-induced hepatotoxicity. Taken together, these findings suggest that Mfn2-mediated mitochondrial fusion is an essential mechanism underlying GCDCA-induced hepatotoxicity in extrahepatic cholestasis.

HMfn2602–757 is a C-terminal truncated form of Mfn2 that no longer has the capacity to induce mitochondrial fusion. Truncated Mfn2-overexpressing cells exhibit a marked enhancement of the mitochondrial membrane potential and the stimulation of ATP production in GCDCA-treated L02 cells. In this study, we observed that the transient transfection of hMfn2602–757 was predominantly localized in the mitochondria and co-localized with mitochondrial markers in immunofluorescence assays. These data agree with a previous study in which hMfn2602–757 was also transiently transfected into HeLa cells, and most of the truncated protein was recruited into mitochondria to directly modulate all of the OXPHOS complexes [11]. Furthermore, our data support additional roles for Mfn2 that extend beyond the regulation of the mitochondrial fusion in GCDCA-induced hepatotoxicity. In line with these findings, Mfn2 plays a secondary role in mitochondrial fusion in several pathophysiological conditions, including diabetes, vascular proliferative disorders, and Charcot-Marie-Tooth type 2A neuropathy [11], [40], [41]. However, as the specific mechanisms involved in the effects caused by the increased activity of the truncated form of Mfn2 remain unknown, we consider two distinct alternatives. First, hMfn2602–757 has been shown to modulate metabolism through the function of the OXPHOS system. Second, hMfn2602–757 may affect the mitochondrial recruitment of Bax or Drp1 during cell death [42]. The two hypotheses are not mutually exclusive, and the details of the mechanisms need further research.

Taken together, our in vitro studies suggest that there may be two biological roles for Mfn2 during the progression extrahepatic cholestasis. On the one hand, Mfn2, which plays significant roles during mitochondrial dysfunction, has been implicated in the control of mitochondrial network. On the other hand, the direct effects of Mfn2 on mitochondrial metabolism do not all depend on its effects as a mitochondrial fusion protein. Specifically, Mfn2 may serve as a therapeutic target for the development of novel treatments to prevent liver damage in patients with extrahepatic cholestasis.
